# α-Hydroxyisocaproic Acid Decreases Protein Synthesis but Attenuates TNFα/IFNγ Co-Exposure-Induced Protein Degradation and Myotube Atrophy via Suppression of iNOS and IL-6 in Murine C2C12 Myotube

**DOI:** 10.3390/nu13072391

**Published:** 2021-07-13

**Authors:** Koichiro Sumi, Misato Sakuda, Kinuyo Munakata, Kentaro Nakamura, Kinya Ashida

**Affiliations:** Food Microbiology and Function Research Laboratories, R&D Division, Meiji Co. Ltd., 1-29-1 Nanakuni, Hachiouji, Tokyo 192-0919, Japan; misato.sakuda@meiji.com (M.S.); kinuyo.munakata@meiji.com (K.M.); kentarou.nakamura@meiji.com (K.N.); kinya.ashida@meiji.com (K.A.)

**Keywords:** α-hydroxyisocaproic acid, TNFα, Interferon-γ, AMPK, ERK, protein synthesis, myotube, atrophy, iNOS, IL-6

## Abstract

There is ongoing debate as to whether or not α-hydroxyisocaproic acid (HICA) positively regulates skeletal muscle protein synthesis resulting in the gain or maintenance of skeletal muscle. We investigated the effects of HICA on mouse C2C12 myotubes under normal conditions and during cachexia induced by co-exposure to TNFα and IFNγ. The phosphorylation of AMPK or ERK1/2 was significantly altered 30 min after HICA treatment under normal conditions. The basal protein synthesis rates measured by a deuterium-labeling method were significantly lowered by the HICA treatment under normal and cachexic conditions. Conversely, myotube atrophy induced by TNFα/IFNγ co-exposure was significantly improved by the HICA pretreatment, and this improvement was accompanied by the inhibition of iNOS expression and IL-6 production. Moreover, HICA also suppressed the TNFα/IFNγ co-exposure-induced secretion of 3-methylhistidine. These results demonstrated that HICA decreases basal protein synthesis under normal or cachexic conditions; however, HICA might attenuate skeletal muscle atrophy via maintaining a low level of protein degradation under cachexic conditions.

## 1. Introduction

α-Hydroxyisocaproic acid (HICA, leucic acid or DL-2-hydroxy-4-methylvaleric acid) is an end-product of the microbial metabolism of leucine (Leu) [1]. Therefore, HICA is found in many fermented foods, including wine, soy sauce, certain cheeses (reviewed by Mero et al. [2]), kimchi [3], and yogurt [4]. The variability of HICA levels in commercial yogurts [4] may reflect the diversity of activities of hydroxyisocaproate dehydrogenase enzymes in the diverse *Lactobacillus* strains used in fermentation [3]. HICA cannot be degraded by many bacterial species, and its production may thus represent a survival strategy for *Lactobacillus*, because HICA displays antibacterial activity [5]. In addition to its impact on bacteria, HICA is generally considered to act as an anticatabolic agent in mammalian tissues [6,7,8]. Therefore, the contributions of HICA to skeletal muscle gain or maintenance have been examined. The impacts on muscle performance could have wide-ranging effects not only on athletic performances but, also, on the quality of life of frail or elderly individuals [9,10,11,12,13].

At present, however, the benefits of HICA for skeletal muscle growth or function remain controversial. In a clinical study, Mero et al. reported that a HICA supplementation of 1.5 g/day led to an increase of lean body mass in soccer players during four weeks of intense training as compared to the placebo [2]. On the other hand, Teixeira et al. recently reported a randomized controlled trial in which HICA supplementation did not show benefits on the fat-free mass, skeletal muscle thickness, or skeletal muscle performance in healthy young men participating in an eight-week resistance-type exercise program [14,15].

Lang et al. reported that an HICA-containing diet promoted the recovery of gastrocnemius muscle weight in rats at 14 days after reloading from a seven-day period of immobilization [16]. Moreover, in both an immobilization period (atrophy) and a late reloading period (hypertrophy), the rates of protein synthesis of animals fed an HICA-containing diet were maintained or were increased relative to the rats fed Leu-fortified or control diets [16]. In principle, the skeletal muscle mass reflects a net balance of skeletal muscle protein synthesis (MPS) and breakdown (MPB) [17], and thus, there is not a contradiction in this relationship between muscle mass and protein synthesis in atrophied and hypertrophied periods. However, the rate of protein synthesis in the non-immobilized gastrocnemius muscle was decreased by the inclusion of HICA in the diet, although those in the heart and liver were not affected [16].

On the basis of these findings, it may be that HICA impacts MPS directly in the healthy state, but it may also ameliorate the decrease of MPS on the way to skeletal muscle atrophy, at least as induced by immobilization. Skeletal muscle immobilization induces skeletal muscle atrophy via increasing oxidative stress and inflammation. For example, oxidative stress mediated by inducible nitric oxide synthase (iNOS) and its product nitric oxide (NO) has been shown to be a critical factor in immobilization-evoked skeletal muscle atrophy in mouse [18]. Additionally, immobilization induces expression of the inflammatory cytokine interleukin-6 (IL-6), and the inhibition of IL-6 expression and/or IL-6 signaling in skeletal muscle ameliorates skeletal muscle atrophy [19,20].

Therefore, we hypothesized that HICA would reduce both the decrease of MPS and the atrophy of skeletal muscles that are evoked by the iNOS- and IL-6-mediated pathways. Since we have obtained the data that HICA is expected to absorb into the blood stream upon oral administration to rats (unpublished data), we investigated the direct effect of HICA on a C2C12 myotube model of muscle atrophy. In this model, the co-exposure of C2C12 myotube cells to tumor necrosis factor α (TNFα) and interferon γ (IFNγ) induces high iNOS and IL-6 expression, resulting in myotube atrophy [21]. We therefore investigated the effect of HICA on MPS and the myotube diameter in healthy or TNFα/IFNγ-exposed myotubes, as well as the propensity of HICA to alter the expression of iNOS and IL-6.

In this study, we demonstrated that HICA decreases MPS at least in part through the activation of adenosine monophosphate-activated protein kinase (AMPK) and the inactivation of extracellular signal-regulated kinase (ERK) in healthy myotubes. We also showed that HICA inhibits the myotube atrophy induced by TNFα/IFNγ co-exposure via the modification of iNOS and/or IL-6 expression.

## 2. Materials and Methods

### 2.1. Materials

DL-HICA was obtained from Sigma-Aldrich (St. Louis, MO, USA). HICA was dissolved in Dulbecco’s modified Eagle’s medium (DMEM) at a concentration of 100 mM, and the solution was neutralized with NaOH and filtered through a 0.22-µm pore polyvinylidene fluoride (PVDF) membrane; this neutralized HICA solution was used to prepare relevant medium samples. Mouse recombinant TNFα and IFNγ were obtained from R&D systems (Minneapolis, MN, USA). Reagents not specifically mentioned were purchased from FUJIFILM Wako Pure Chemical Corporation (Osaka, Japan) or Sigma-Aldrich.

The following antibodies were obtained from Cell Signaling Technology (CST, Beverly, MA, USA): phospho-AMPKα, total-AMPKα, phospho-ACC, total-ACC, phospho-ULK1, total-ULK1, total-p70S6K, phosopho-4E-BP1, total-4E-BP1, phospho-ERK1/2, total-ERK1/2, phospho-p38MAPK, total-p38MAPK, phospho-eEF2, total-eEF2, phospho-STAT3, total-STAT3, phospho-p65NFκB, total NF-κB, and β-tubulin. Phospho-p70S6K, iNOS, and GAPDH antibodies were obtained from Affinity Biosciences (Cincinnati, OH, USA), BD Biosciences (Franklin Lakes, NJ, USA), and GenTex (Irvine, CA, USA), respectively. Atrogin-1 and muscle RING-finger protein-1 (MURF-1) antibodies were obtained from Abcam (Cambridge, UK).

### 2.2. Cell Culture

A mouse myoblast cell line (C2C12) was obtained from ATCC (Manassas, VA, USA). C2C12 myoblasts were grown in DMEM containing 10% fetal bovine serum and 1% penicillin and streptomycin (PenStrep) (growth medium (GM)). For myotube differentiation, C2C12 myoblasts were seeded in gelatin-coated six-well cell culture plates or eight-well chamber slides (idibi GmbH, Gräfelfing, Germany) and grown to subconfluence in GM, and then, GM was replaced with a differentiation medium (DM), which consisted of DMEM containing 2% horse serum and 1% PenStrep. The mediums were changed every 2 days, unless otherwise noted in the method.

### 2.3. Scheme of Cell Culture Experiments

Details of the time courses of each set of experiments are provided in their respective figure legends. In brief, for the investigation of responses of normal myotubes to HICA, C2C12 were fully differentiated by a 4- to 5-day incubation in DM prior to each experiment. For investigation of the effects of HICA on myotube atrophy, TNFα (3 ng/mL) and IFNγ (7.5 ng/mL) were added to the medium three days after the addition of DM. The cytokine concentrations were lower than those used in previous reports [21,22], but in our laboratory, they were adequate to induce myotube atrophy and to increase the expression of iNOS and IL-6.

### 2.4. Capillary Electrophoresis-Based Immunoassay and Western Blotting

The expression of proteins other than atrogin-1 and MURF-1 was analyzed using an automated capillary electrophoresis immunoassay system (Wes, Protein Simple, CA, USA), as described previously [23]. To extract the whole protein from C2C12 myotubes, we added an ice-cold Laemmli sample buffer (63-mM Tris-HCl, pH 6.8, 2% sodium dodecyl sulfate, 50-mM dithiothreitol, 10% glycerol, 33-mM sodium fluoride, 16-mM β-glycerophosphate, 1.3-mM sodium orthovanadate (V), and 1% Sigma protease inhibitor cocktail P8340) to cells and homogenized them by sonication. For each antibody employed, we confirmed the presence of a detectable peak with the appropriate specificity and the appropriate apparent molecular weight. The phosphorylation levels were calculated by dividing the phosphorylation signal by that of the corresponding total protein. 

The protein expression of atrogin-1 and MURF-1 were analyzed by traditional Western blotting. Samples were extracted as described for the capillary electrophoresis immunoassays. Following the separation by SDS-PAGE on 6% polyacrylamide gels, proteins were transferred electrophoretically to a PVDF membrane using a semi-dry blotting system (Bio-Rad, Hercules, CA, USA). Membranes were blocked with Can Get Signal blocking reagent (TOYOBO, Osaka, Japan) for 1 h at room temperature and then incubated with atrogin-1 or MURF-1 antibodies in Can Get Signal solution 1 (TOYOBO) overnight at 4 °C. Signals of the proteins of interest were detected by ECL Prime (GE Healthcare, Chicago, IL, USA) and captured by ChemDoc densitometry (Bio-Rad) after incubation with horseradish peroxidase-conjugated anti-rabbit IgG antibodies (CST). After detection, antibodies were stripped with Restore Plus reagent (Thermo Fischer, Waltham, MA, USA), and membranes were reblotted as above to detect GAPDH. 

Capillary electrophoresis immunoassays and Western blotting were performed at least twice for each protein of interest, and the results were averaged. We used β-tubulin as a loading control for the normal myotube experiments, but we changed to GAPDH as the housekeeping gene for the experiments involving cytokine exposure, because we noticed that β-tubulin expression was affected by cytokine exposure.

### 2.5. Protein Synthesis Assays

Basal protein synthesis was measured by metabolically labeling alanine (Ala) with deuterium oxide (D_2_O), as described previously [24]. In brief, cells were harvested 16 to 24 h after the addition of D_2_O or H_2_O (as an unlabeled control) into the medium at a final concentration of 4%. Two wells of unlabeled cells were set for each evaluation sample group. Harvested cells were washed and resuspended in 6-N HCl and hydrolyzed by 1-h incubation at 150 °C under decompression. The distribution of the Ala isotopomer mass was measured by a 2,3,4,6-tetra-O-acetyl-β-D-glucopyranosyl isothiocyanate (GITC; Mw:389) derivatization method, followed by a liquid chromatography tandem mass spectrometry (LC-MSMS) analysis, as described [25]. The M0, M1, and M2 mass isotopomers of GITC-Ala (mass-to-charge ratio (*m/z*) = 478.5, 479.5, and 480.5, respectively) were analyzed by positive electrospray ionization (ESI) selected ion monitoring. The excess molar enrichment of M + 1 GITC-Ala (EM_1_ (%)) was determined using Formula (1):

EM_1_ = [(M1/(M0 + M1 + M2)) _sample_ − averaged (M1/(M0 + M1 + M2)) _unlabeled control_] × 100 (1)

The asymptotic maximum EM_1_ (EM_1,max_) of a tissue protein compartment can be calculated as described previously [26] using the precursor pool (p) set as the deuterium enrichment in the medium (4 atom%) and the (*n*) set as 4 (i.e., the number of exchangeable bonds in Ala). (p) was set at 4 atom% according to our preliminary investigation that the water D/H% obtained from the medium was maintained at approximately 4 atom% from 4% D_2_O addition to 24 h after that. The fraction of protein (f) that was newly synthesized during the labeling period (t) was calculated as the ratio of the measured EM_1_ to EM_1,max_. The fractional synthesis rate constant (k) was calculated using a rise-to-plateau kinetics formula (Equation (2)) with individual (f) or (EM_1_) and deuterium-labeling time values (t) substituted as described previously [24,26].

f_(t)_ = f_max_ × (1 − e^−kt^) or EM_1,(t)_ = EM_1,max_ × (1 − e^−kt^) (2)

The basal fractional synthesis rate (FSR) was expressed by converting the rate constant, k, to the rate of protein synthesis in units of % per h. 

### 2.6. Immunofluorescence

Cells were fixed with 4% paraformaldehyde in phosphate-buffered saline (PBS) for 15 min at room temperature and were washed 3 times with PBS. Fixed cells were blocked with Tris-buffered saline (TBS) containing 3% bovine serum albumin (fraction V) and 0.2% Triton X-100 for 30 min at room temperature. Myosin heavy-chain antibodies (MF20, Affymetrix, Santa Clara, CA, USA) were diluted 1:250 in Signal Booster Immunostain Solution F (Beacle Inc., Kyoto, Japan) and were incubated with cells for 2 h at 37 °C. After the cells were washed with TBS with 0.1% Tween 20 (TBS-T), Alexa-Fluor 488-conjugated anti-mouse IgG2b antibodies (Invitrogen, Carlsbad, CA, USA) and 1-mg/mL Hoechst 33258 solution (DOJINDO, Kumamoto, Japan) were diluted 1:2000 and 1:10,000 in Signal Booster Solution F and incubated with cells for 1 h at room temperature. After cells were washed 3 times in TBS-T, cells were covered with antifade mounting medium (idibi GmbH) and observed by fluorescent microscopy using GFP and DAPI filters (BZ-X810, KEYENCE Corporation, Osaka, Japan). Several microscopic images (3 × 3) were gathered per chamber and were combined into one picture using Image Analyzer application software (KEYENCE Corporation). 

**Figure A1:**
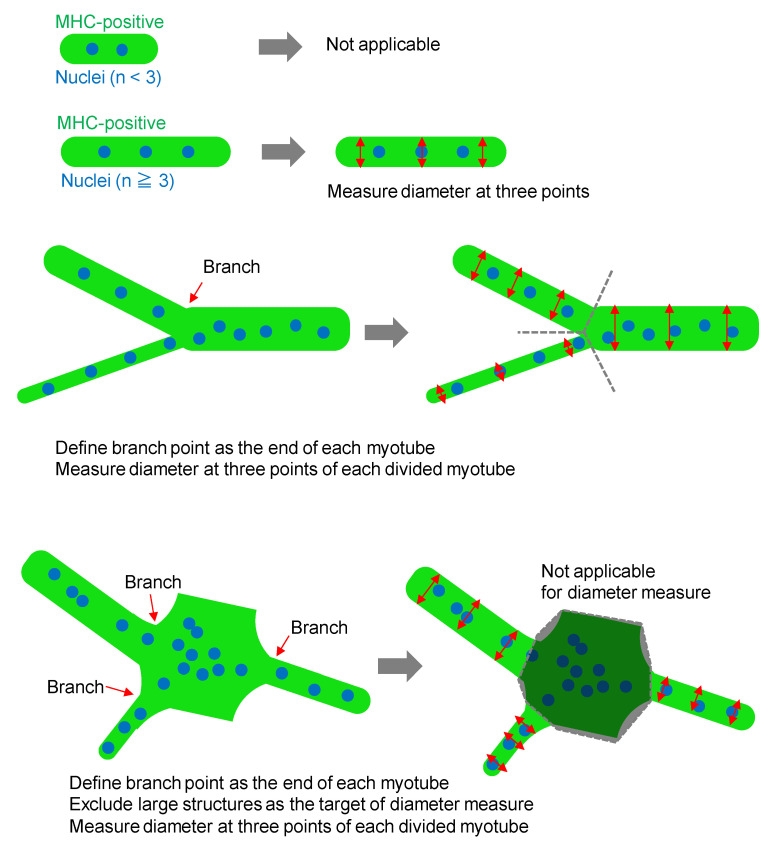
Schematic diagram of a typical measurement of myotube diameters. Myotubes were defined as myosin heavy-chain (MHC)-positive and multinuclear (≥3 nuclei) cells, and the myotubes for which both ends (edge or branched points) were visible in the composite image were analyzed. Diameters of the myotubes were measured at three positions (near both ends and center region; red double-headed arrow) using ImageJ software and averaged. Large structures containing connections among many myotubes were excluded from the diameter measurements, because the tube shapes could not be defined (see the lower-most region of the figure).

Myotubes were defined as myosin heavy-chain (MHC)-positive and multinuclear (≥3 nuclei) cells, and the myotubes for which both ends (edge or branched points) were visible in the composite image were analyzed. Diameters of the myotubes were measured at three position (near both ends and the center region) using Image J software (ver. 1.52p, Wayne Rasband, National Institutes of Health, Bethesda, MD, USA) and averaged. Large structures that contained many connected myotubes were excluded from the diameter measurements, because the tube shapes could not be defined. The typical manner of diameter measuring is represented in Figure A1. To quantify the fusion indices, the percentages of nuclei in MHC-positive cells with two or more nuclei per cell in each composite image (*n* > 5000) were measured using ImageJ software.

### 2.7. Analysis of Culture Medium Components

NO_2_ and NO_3_ concentrations in the medium, which reflect NO synthesis, were measured with a commercially available kit (DOJINDO). Medium IL-6 was measured using a commercial ELISA kit (BioLegend, San Diego, CA, USA). The release of 3-methyl histidine (3-MeHis), which is contained in myosin and actin and is not recycled for use in protein synthesis [27], into the medium reflects the degradation of myotube proteins [28], and it was measured as described [23]. Briefly, supernatants obtained by centrifuging after protein precipitation with 3% trichloroacetic acid were filtered, and the 3-MeHis content was measured by LC-MSMS with an Intrada amino acid column (Intact Corp., Kyoto, Japan).

### 2.8. Statistics

All results were shown as the means ± standard deviation (SD). For comparison of the results between two groups, a Student’s *t*-test or Welch test was carried out. For comparisons among multiple groups, the significance of the effects associated with the test groups was analyzed by one-way ANOVA, and differences between all pairs or between the control group and other groups were tested by a Tukey–Kramer or Dunnett post hoc test, respectively. For analyzing any two main effects, such as time, cytokine, and/or HICA treatment effects, we used a two-way ANOVA, and a post hoc simple main effect test was performed if an interaction between any two main effects was significant. The significance of all the tests was set at *p* < 0.05. All analyses were performed with JMP 11 software (SAS Institute Inc., Cary, NC, USA).

## 3. Results

### 3.1. Effects of HICA on Normal C2C12 Myotubes

#### 3.1.1. Dose-Dependent Effects of HICA on p70S6K and AMPK Phosphorylation

**Figure 1:**
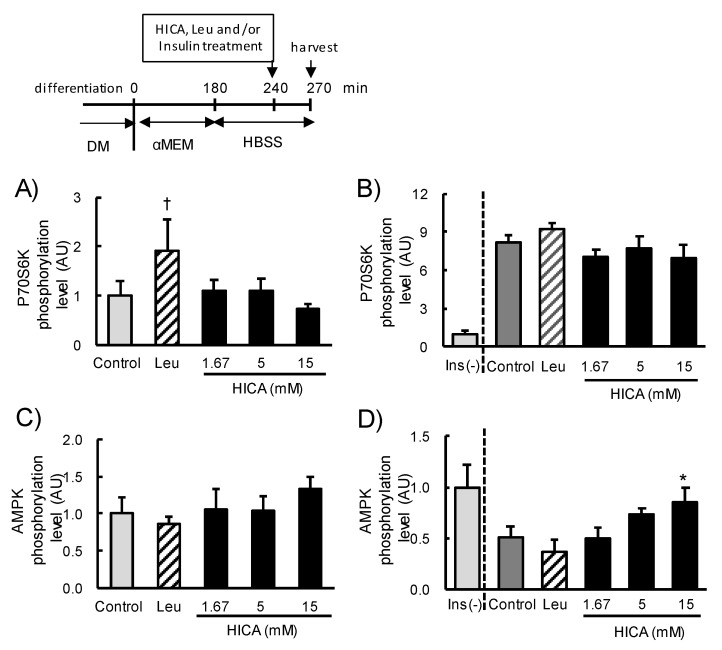
The effects of HICA treatment on the phosphorylation of p70S6K and AMPK. The phosphorylation level of p70S6K 30 min after Leu or HICA treatments without (**A**) or with (**B**) 2-µM insulin (Ins). The phosphorylation level of AMPK 30 min after Leu or HICA treatment without (**C**) or with (**D**) 2-µM insulin. (**B**,**D**) The levels in untreated cells without insulin are shown in the left-most bar as a reference. The time course of this set of experiments is shown in the upper panel. DM: differentiation medium, αMEM: α modified Eagle’s medium (for serum starvation), and HBSS: Hank’s balanced salt solution (for complete starvation). Data are displayed as the means ± SD, *n* = 4 for each group in all panels. ^†^ *p* < 0.1 vs. the nontreated control group. * *p* < 0.05 vs. the insulin-treated control group.

To examine whether HICA supplementation correlates with the acceleration of protein synthesis through the mTORC1 signaling pathway, we analyzed the phosphorylation of p70S6K, which is a major downstream target of mTORC1 [29]. Phospho-p70S6K was quantified at 30 min after exposure to 1.67-mM, 5-mM, or 15-mM HICA or 5-mM Leu following 4 h of serum starvation (incubation with αMEM) and an additional 30 min of complete starvation (incubation with HBSS), as described in a previous report [30]. The phosphorylation of p70S6K in Leu-exposed cells was apparently higher than in cells of the control group, but the difference was not statistically significant (*p* < 0.1). The levels in all HICA-exposed cells were the same or slightly lower than the control (Figure 1A). In a previous study, HICA was supplied to rats via mixing into refined feed [16]; therefore, the additive effect of HICA with insulin was also investigated. Here, HICA at all doses did not further increase the phosphorylation of p70S6K as increased by 2-µM insulin (Figure 1B). These results suggest that HICA does not accelerate protein synthesis via the mTORC1 pathway in normal C2C12 myotubes.

Since we noticed that the phosphorylation levels of p70S6K were relatively low in the HICA-exposed groups (Figure 1A,B), the phosphorylation levels of AMPK, which is one of the negative regulators of the mTORC1 pathway, were examined. HICA supplementation was accompanied with an increased AMPK phosphorylation in a dose-dependent manner, and AMPK phosphorylation in the 15-mM HICA exposure group was significantly higher than that of the control group in the presence of insulin (*p* < 0.05) (Figure 1D), although there was no significant increase in the absence of insulin (Figure 1C). These results suggest that 15-mM HICA exposure might negatively regulate protein synthesis in normal C2C12 myotubes, and we therefore used a 15-mM dose of HICA in subsequent investigations.

#### 3.1.2. The Effects of HICA on Signaling Pathways That Regulate Protein Synthesis

We examined the effect of 30 min of 15-mM HICA treatment on the mTORC1 and AMPK signaling pathways. The treatment with HICA occurred after 3 h of serum starvation and a subsequent 1 h of serum and amino acid starvation. These conditions were used previously to evaluate the AMPK agonist 5-aminoimidazole-4-carboxamide-1-β-D-ribofuranoside (AICAR) [31]; the use of similar conditions allowed us to compare the effects on AMPK of HICA to those of AICAR. 

**Figure 2:**
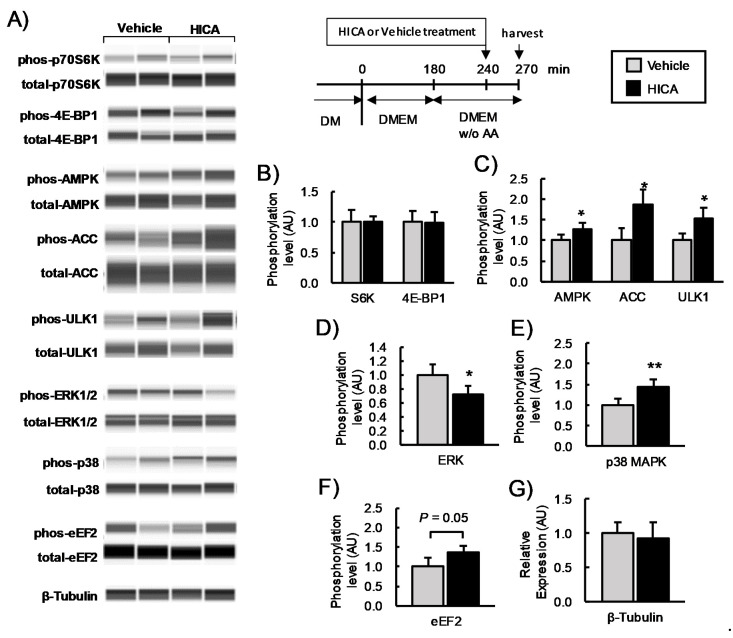
The effects of HICA on the intracellular signaling pathways. A typical image for a capillary immunoassay is shown (**A**). The phosphorylation levels of (**B**) p70S6K and 4E-BP1; (**C**) AMPK, ACC, and ULK1; (**D**) ERK1/2; (**E**) p38MAPK; and (**F**) eEF2 are shown. The phosphorylation is normalized to the total protein expression. The β-tubulin content in the lysate was measured as a loading control (**G**). The time course of these experiments is shown in the upper region. DM: differentiation medium, DMEM: Dulbecco’s modified Eagle’s medium, and *w/o* AA: without amino acids. Data are displayed as the means ± SD, and *n* = 4 for each group in all bar graphs. * *p* < 0.05 and ** *p* < 0.01 vs. the vehicle-treated group.

HICA significantly increased the phosphorylation of AMPK and its downstream target acetyl-CoA carboxylase (ACC) [32] and the phosphorylation of Ser555 of UNC-51-like kinase (ULT1) [33]. The HICA treatment did not affect the phosphorylation of p70S6K or 4E-BP1, which are other mTORC1 targets [29] (Figure 2A–C). Additionally, we examined the phosphorylation of two mitogen-activated protein kinases (MAPK), ERK1/2 and p38MAPK. HICA significantly decreased the phosphorylation of ERK1/2 (*p* < 0.05) but significantly increased that of p38MAPK (*p* < 0.01) (Figure 2A,D,E). The phosphorylation of eukaryotic elongation factor 2 (eEF2), whose translation elongating activity is inhibited by excess phosphorylation [34], was apparently higher in the cells treated with HICA as compared to the vehicle treatment, but this difference did not reach statistical significance (*p* < 0.1) (Figure 2A,F). Expression of the housekeeping molecule β-tubulin was similar in HICA- and vehicle-treated cells (Figure 2A,G). These findings suggested that HICA negatively regulates protein synthesis through multiple pathways, including AMPK and p38 MAPK activation and ERK inhibition, in normal C2C12 myotubes.

#### 3.1.3. The Effects of HICA on Fractional Protein Synthesis Rates in Normal C2C12 Myotubes and Myoblasts

**Figure 3:**
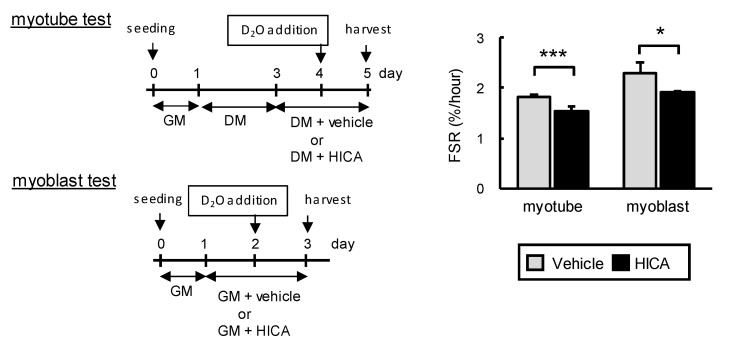
Basal fractional protein synthesis rate (FSR) of the vehicle- or HICA-treated myotubes and myoblasts. The time course of this set of experiments is shown on the left. GM: growth medium and DM: differentiation medium. Means ± SD, both group *n* = 6 or 4 in the myotubes or myoblasts, respectively. * *p* < 0.05 and *** *p* < 0.001 vs. each vehicle-treated group.

We investigated the effect of HICA on the FSR of normal C2C12 myoblasts and differentiated C2C12 cells (myotubes) by a method involving metabolic labeling with D_2_O (see Figure 3). Differentiated C2C12 cultures, which contain myotubes and nondifferentiated stationary cells, are known to exhibit a decreased FSR compared to proliferative myoblasts [24]. Similarly, in the present experiment, we observed that FSRs of the vehicle-treated myotubes and myoblasts were approximately 1.8% and 2.3%/h, respectively (Figure 3), whereas the FSRs of the HICA-treated myotubes and myoblasts were approximately 1.5% and 1.9%/h, respectively. These FSRs were significantly lower than those of the corresponding vehicle-treated myotubes or myoblasts (Figure 3). These results indicate that a HICA treatment results in a deceleration of FSR of C2C12 myotubes and myoblasts, at least under normal conditions.

### 3.2. HICA Effects on Cachexic C2C12 Myotubes

#### 3.2.1. The Effects of HICA on the Diameter of Cachexic Myotubes

**Figure 4:**
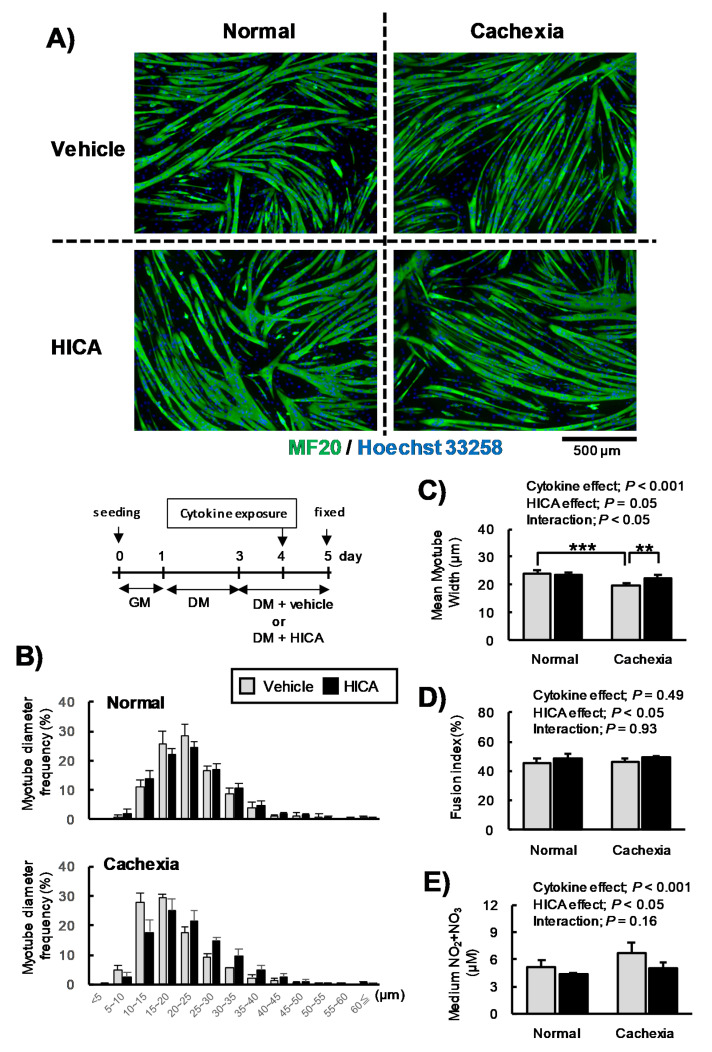
Effects of HICA on myotube atrophy. Typical myosin- (green; MF20) and nuclear (blue; Hoechst 33258)-stained myotubes are shown (**A**). Distributions of the diameters of the tubes in the vehicle- and HICA-treated groups under normal (upper panel) or cachexic conditions (lower panel) are shown (**B**). Mean myotube diameters are shown (**C**), the fusion indices are shown (**D**), and the nitric oxide secretion (expressed as µM total NO_x_ in the medium) is shown (**E**). The time course of these experiments is shown in the upper region (**B**). GM: growth medium and DM: differentiation medium. Data are presented as the means ± SD for each group (*n* = 4). The diameter of the 218–323 myotubes per each chamber were measured. The results of the two-way ANOVAs are shown in the upper region (**C**,**D**). ** *p* < 0.01 and *** *p* < 0.001 per the results of a post hoc simple main effect test.

**Figure A2:**
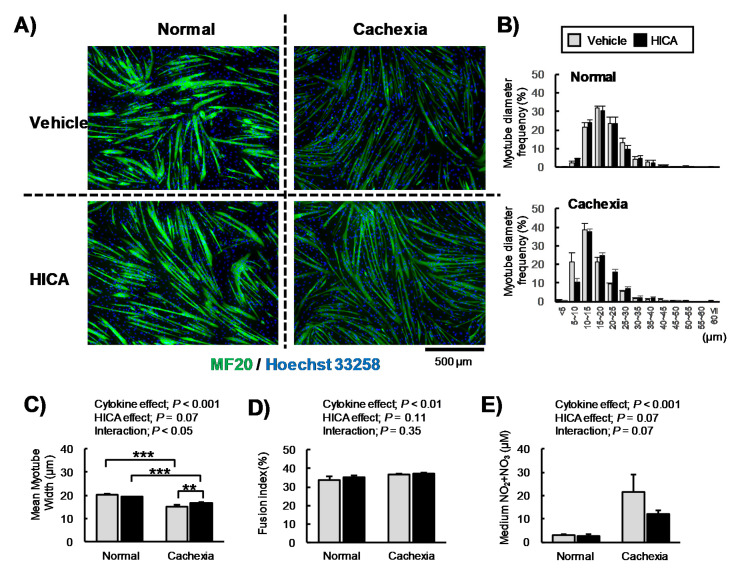
A series of experiments similar to those displayed in Figure 5. We re-evaluated the effect of the HICA pretreatment on cachexic myotubes at relative early stages of differentiation as compared to the experiment shown in Figure 5. Typical myosin- (green; MF20) and nuclear (blue; Hoechst 33258)-stained myotubes are shown (**A**). the distributions of the diameters of the vehicle- and HICA-treated groups under normal (upper panel) and cachexic conditions (lower panel) are shown (**B**). The mean myotube diameter (**C**), fusion index (**D**), and nitric oxide secretion into the medium is expressed as the µM total NO_x_ (**E**). The time course of these experiments is similar to that shown in Figure 5, but this series of experiments involved a relatively low differentiation efficiency (compare the fusion indices in Figure 5 to those in this figure). Data are presented as the means ± SD for each group (*n* = 3). The diameters of the 286–344 myotubes per each chamber were measured. The results of the two-way ANOVAs are shown in the upper region (**C**–**E**). ** *p* < 0.01 and *** *p* < 0.001 per the results of a post hoc simple main effect test.

The condition cachexia is mimicked by co-exposure to TNFα and IFNγ, and it induces decreasing of the diameters of C2C12 myotubes, as seen in skeletal muscle atrophy [21]. These phenomena are caused, at least in part, by the over-secretion of NO [21,22]. Therefore, we examined the effects of HICA on the myotube diameter and NO secretion under a cachexic condition. Immunostained myotubes (Figure 4A), the average and distribution of myotube diameters (Figure 4B,C), the fusion index (Figure 4D), and the media NOx concentrations (Figure 4E) at 16 h after exposure to cytokines following a pretreatment with HICA or the vehicle are shown in Figure 4. To further investigate these results, we conducted another similar series of experiments, except that the myotube differentiation conditions were established earlier relative to the experiments described in Figure 4 (Figure A2).

Two days of HICA treatment did not affect the distribution of myotube diameters under normal conditions (Figure 4B), whereas HICA negatively regulated the protein synthesis, as described above. Interestingly, however, the pretreatment with HICA appeared to suppress the increase in thin fibers, especially those fibers with diameters of less than 15 µm, that was caused by cytokine exposure (Figure 4B). The interaction between cytokine exposure and HICA treatment on the average myotube diameter was significant (*p* < 0.05), and cytokine exposure significantly decreased the myotube diameters in the vehicle-treated group (*p* < 0.001) but did not affect the diameters in the HICA-treated group (*p* > 0.05). In cells treated with TNFα/IFNγ, the HICA treatment led to a significant increase in the myotube diameters relative to the vehicle treatment (*p* < 0.01) (Figure 4C). The fusion indices of vehicle- or HICA-treated myotubes under normal or cachexic conditions were 45%, 48%, 46%, or 49%, respectively, and HICA significantly increased the fusion index (HICA main effect; *p* < 0.05) (Figure 4D). Moreover, HICA significantly decreased NOx secretion (HICA main effect; *p* < 0.05), whereas TNFα/IFNγ co-exposure significantly increased NOx secretion (cytokine main effect; *p* < 0.001) (Figure 4D). Therefore, the increased NOx concentrations that were induced by cytokine exposure were attenuated by the HICA pretreatment.

According to the results shown in Figure 4, we needed to consider the possibility that HICA accelerates myotube differentiation and that the effects of HICA on myotube diameters under cachexic conditions depend on this acceleration. However, in another series of similar experiments, the diameters of HICA-pretreated myotubes were found to be significantly higher than those of vehicle-treated myotubes under the cachexic condition, even though the diameters of myotubes from both TNFα/IFNγ exposure groups were significantly decreased relative to the respective normal groups (Figure A2B,C). The fusion indices were not increased by the HICA treatment (HICA main effect; *p* = 0.11) but were affected by cytokine exposure (cytokine effect; *p* < 0.01) (Figure A2D). In addition, the trends of NO production induced by cytokine exposure were attenuated by HICA pretreatment (Figure A2E). These results suggest that HICA suppresses the myotube atrophy caused by exposure to TNFα and IFNγ via the inhibition of NO secretion.

#### 3.2.2. The Effects of HICA Pretreatment on Acute Cytokine Signaling

**Figure 5:**
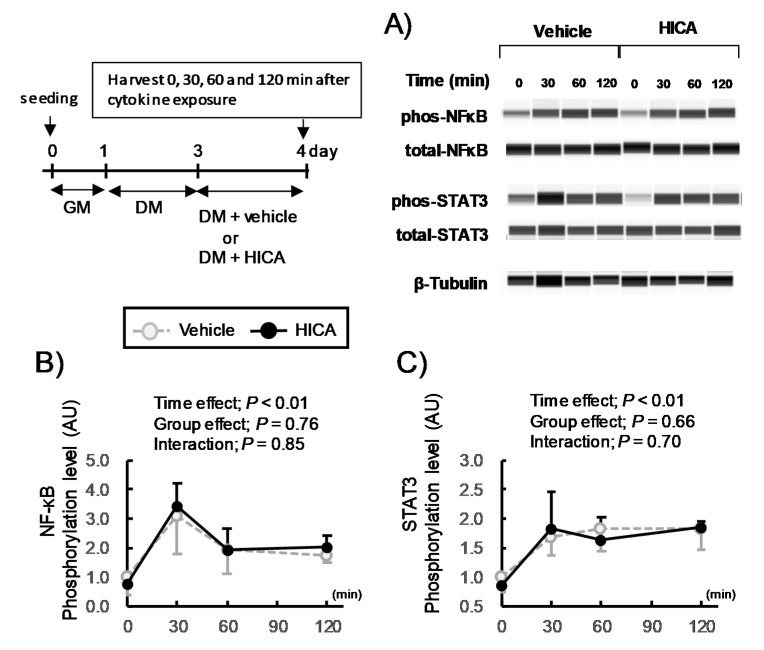
Effects of HICA on the acute cytokine signaling events. A typical image for capillary immunoassays is shown (**A**). The phosphorylation levels of (**B**) STAT3 and (**C**) p65NFκB in the whole-cell lysate were quantified. The phosphorylation levels were normalized to the total amount of that protein. β-Tubulin was used as a loading control (**A**). The time course of this set of experiments is shown in the left upper panel. GM: growth medium and DM: differentiation medium. Data are presented as the means ± SD (*n* = 3). The results of the one-way ANOVAs are shown in the upper regions (**B**,**C**).

The addition of both TNFα and IFNγ to C2C12 myotubes induces the translocation of both STAT3 and NF-κB into the nucleus, resulting in the overexpression of acute-inflammatory proteins, including iNOS [21]. Importantly, then, the phosphorylation status of STAT3 and NF-κB is indicative of the activation of their translocation to the nucleus and the acceleration of the transcription of their target genes [21,35,36]. Therefore, we examined the effects of HICA pretreatment on the phosphorylation of STAT3 and p65 NF-κB that is induced by the coaddition of TNFα and IFNγ (Figure 5). The phosphorylation level of STAT3 was over three-fold higher 30 min after the cotreatment with TNFα and IFNγ, and phosphorylation was sustained at a level approximately two-fold higher than that of the time 0 samples until 120 min after cytokine co-exposure. Similarly, the phosphorylation levels of p65 NF-κB remained elevated 30 to 120 min after cytokine co-exposure. The effects of the HICA pretreatment were not significant on the phosphorylation of either STAT3 or NF-κB after the cytokine addition (Figure 5B,C, the time main effects were significant, but the group main effect or interaction were not significant in both STAT3 and NF-κB). These findings suggest that the positive effects of HICA on cachexic myotubes do not result from the modification of acute STAT3 or NF-κB signaling.

#### 3.2.3. The Effects of HICA Pretreatment on Protein Synthesis and Expression of Inflammatory Proteins 

**Figure 6:**
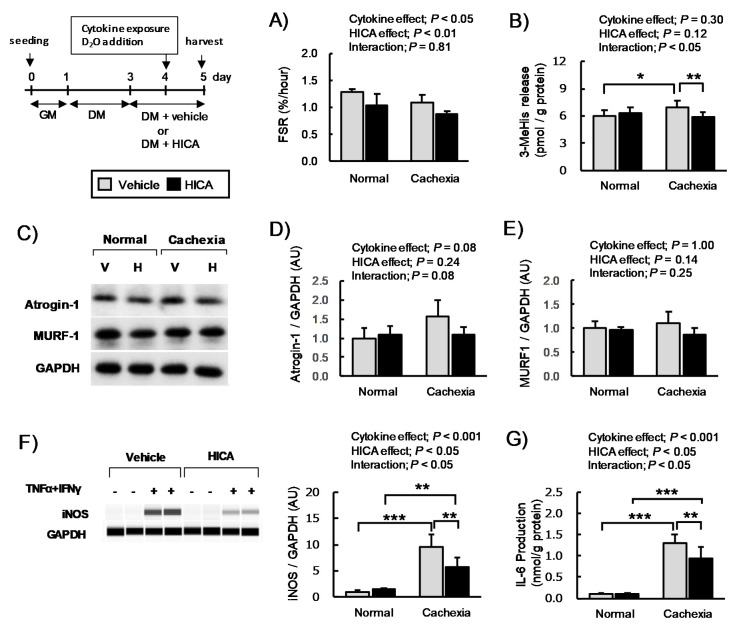
Effects of HICA on the protein synthesis and production of inflammatory proteins. The basal fractional protein synthesis rate (**A**); 3-MeHis concentration in the medium (**B**); relative expression of atrogin-1, MURF1, and iNOS (**C**–**F**); and IL-6 production (**G**) of vehicle- or HICA-treated myotubes under normal or cachexic conditions. The time course of this set of experiments is shown in the upper left region. GM: growth medium and DM: differentiation medium. A typical image for Western blotting is shown (**C**). V: vehicle and, H: HICA (**C**). A typical image for a capillary immunoassay is shown in the left panel (**F**). Atrogin-1, MURF-1, and iNOS expression in the myotubes is represented as the intensity relative to that of GAPDH, which was used as a housekeeping protein. Data are presented as the means ± SD (*n* = 4 or 6 in (**A**,**D**,**E**) or (**B**,**F**,**G**), respectively). The results of the two-way ANOVA are shown in the upper regions (**A**,**B**,**D**–**G**). * *p* < 0.05, ** *p* < 0.01, and *** *p* < 0.001 per the results of a post hoc simple main effect test.

AICAR attenuates the myotube atrophy and downregulation of basal protein synthesis caused by TNFα/IFNγ co-exposure [37]. We expected that the effects of HICA on myotube FSR both in the normal and cachexic conditions would be similar to those previously found for AICAR. In this study, however, both main effects of cytokine and HICA were significant, but the interaction was not significant (Figure 6A). Therefore, HICA pretreatment decreased myotube FSR in both the normal and cachexic conditions. 

Generally, skeletal muscle hypertrophy and atrophy are prescribed by a net balance of MPS and MPB [17]. Therefore, we investigated the secretion of 3-MeHis, which reflects MPB, and we examined the expression of atrogin-1 and MURF-1, which are two of the major muscle-specific F-box-type E3 ubiquitin ligases that accelerate MPB via the ubiquitin–proteasome system [38,39]. Here, the interactions between the main effects of cytokine exposure and HICA treatment were significant (*p* < 0.05). In addition, while cytokine exposure significantly increased 3-MeHis secretion in the vehicle-treated group (*p* < 0.05), it did not affect 3-MeHis secretion in the HICA-treated group (*p* > 0.05). Thus, 3-MeHis secretion in HICA-treated myotubes was significantly lower than in vehicle-treated myotubes under TNFα/IFNγ co-exposure conditions (*p* < 0.01) (Figure 6B). 

Moreover, with regard to atrogin-1 and MURF-1 expression, we found that atrogin-1 expression was increased upon cytokine exposure, but this change did not reach the level of statistical significance (cytokine main effect; *p* = 0.08), and the expression was increased slightly upon HICA pretreatment (relative expression of the cachexia-vehicle and cachexia-HICA groups; 1.6 ± 0.4 vs. 1.1 ± 0.2, cytokine x HICA interaction; *p* = 0.08), although, in neither case did the HICA effect on the protein expression reach significance. The expression of MURF-1 was not affected to a significant effect by the treatment with cytokines or with HICA (Figure 6E). These results suggest that HICA cannot recover the decreased FSR induced by TNFα/IFNγ co-exposure, but it does attenuate increases in myotube protein degradation by inhibiting the increased activity of the ubiquitin–proteasome system, at least in part.

Myotube atrophy induced by TNFα/IFNγ co-exposure is ameliorated by a low dose of the translation inhibitor pateamine A through suppression of the increase of iNOS [40]. In addition to increasing iNOS, TNFα/IFNγ co-exposure also dramatically increases IL-6. Therefore, we examined the effects of HICA on IL-6 production and iNOS expression both under normal and cachexic conditions. The interactions between the main effect of cytokine exposure and HICA treatment were significant in regard to iNOS expression (Figure 6F). The cytokine exposure dramatically increased the iNOS expression in both the vehicle- and HICA-treated myotubes, but under cachexic conditions, the iNOS expression upon treatment with HICA was significantly lower than with the vehicle. The effects of TNFα/IFNγ co-exposure and HICA treatment on IL-6 production tended to be similar to those on iNOS (Figure 6G). These results suggest that HICA pretreatment might inhibit cytokine-induced acute increases of iNOS expression and IL-6 production via the suppression of basal protein synthesis.

## 4. Discussion

In this study, we tested our hypothesis that HICA would attenuate the myotube atrophy that accompanies a decrease of protein synthesis using an in vitro cachexia model evoked by a co-exposure to TNFα and IFNγ. However, we obtained conflicting findings. On the one hand, we confirmed the finding that HICA attenuates myotube atrophy. On the other hand, we found that HICA decelerates protein synthesis in the normal and cachexic conditions. The following sections discuss the interesting phenomena found in this study.

Our results on normal C2C12 myotubes clearly showed that HICA negatively regulates protein synthesis. Protein synthesis, called translation, is a dynamic physiological event in which amino acids are sequenced and polymerized using mRNA as a template [41]. Protein synthesis is carried out by the collaboration of various molecules, including 4EBP-1, which is involved in translation initiation, eEF2, which is involved in translation elongation, and p70S6K, which is involved in both of these processes that influence protein synthesis [29,41]. Since the HICA treatment tended to induce eEF2 phosphorylation rather than to suppress p70S6K or 4E-BP1 phosphorylation, HICA might downregulate MPS through inhibiting translational elongation independently of mTORC1 signaling-mediated suppression. 

The AMPK agonist AICAR decreases protein synthesis both by downregulating p70S6K and 4E-BP1 phosphorylation and by upregulating eEF2 phosphorylation in C2C12 myotubes [31]. HICA modified the phosphorylation of these signaling molecules in a manner similar to AICAR but inversely with respect to ERK1/2, whose phosphorylation levels are increased by AICAR [31]. The phosphorylation of eEF2 is catalyzed by the eEF2 kinase (eEF2K), which is a downstream target of both p70S6K and p90RSK [42]. Therefore, HICA might decrease p90RSK phosphorylation through deactivation of its direct regulator ERK1/2, resulting in an increase of eEF2 phosphorylation. 

In contrast with ERK, the phosphorylation of p38MAPK was increased by the HICA treatment in this study. In interval-exercised muscle, the p38 MAPK pathway, which is activated by several stimuli such as growth factors, inflammation, and environmental stress [43], correlates with activation of the AMPK pathway and may induce biogenesis of the mitochondria, resulting in an increasing oxidative capacity [44]. Therefore, further studies to examine the effects of HICA on mitochondrial contents, myofiber-type distribution, glucose transport, metabolic flux, and other metabolic factors are warranted.

In the cachexic myotube model that involved co-exposure to TNFα and IFNγ, we found that HICA suppressed myotube atrophy but did not affect the decrease in protein synthesis. Although it is necessary to consider that the effects of the 15-mM HICA treatment on the phosphorylation of molecules in the AMPK pathway were much weaker than those of the 0.5-mM AICAR treatment used in previous reports [31,37] (our unpublished data), these results suggest that HICA acted to improve myotube atrophy by a different mechanism than AICAR used [37].

Nitric oxide has an important role in muscle hypertrophy, especially hypertrophy mediated by resistance exercise [45], but its overload attenuates protein synthesis, resulting in myotube wasting [46]. TNFα and IFNγ co-exposure drastically increases NO secretion from C2C12 myotubes via iNOS overexpression induced by the cooperative signaling of STAT3 and NF-κB [21]. We found that HICA inhibited the enhancement of NO secretion and iNOS expression induced by TNFα and IFNγ co-exposure, but HICA supplementation seemed not to affect the STAT3 or NF-κB signaling. Moreover, we preliminarily tested the effect of the iNOS inhibitor W-1400 on the cachexic myotube, but its phenotype did not resemble that of HICA (data not shown). Similar to the AMPK-activating effect compared to AICAR, the inhibited effects on iNOS and NO of HICA were not strong compared to W-1400; thus, we believe that our observed phenomena with HICA pretreatment was an assembly of small effects such as iNOS inhibition. Our results led us to speculate that HICA inhibited the cytokine-induced accumulation of inflammatory proteins such as iNOS by simply suppressing basal protein synthesis. Indeed, the production of IL-6, which is one of the major inflammatory proteins secreted from skeletal muscle [47] and which is markedly increased by TNFα and/or IL-1β [48], as well as TNFα/IFNγ co-exposure [21] in myocytes, was decreased by a pretreatment with HICA prior to cytokine stimulation in this study. Prolonged IL-6 exposure leads to skeletal muscle atrophy accompanied by enhanced catabolism, mainly of the myofibril protein [49,50], whereas short-term IL-6 elevation leads to increased protein synthesis [51].

Additionally, we found that HICA pretreatment attenuated the cytokine-induced enhancement of 3-MeHis secretion and atrogin-1 expression. These results suggest that HICA might inhibit the myotube protein degradation induced by iNOS and/or IL-6 at least in part via suppression of the ubiquitin–proteasome system. This inhibition might be a mechanism whereby HICA inhibits myotube atrophy, because the ubiquitin–proteasome system is an important contributor to the skeletal muscle and myotube atrophy induced under cachexic conditions [52]. Moreover, exercise, especially severe eccentric contractions, potentially induces protein degradation, accompanied by NO and IL-6 production [53,54,55]. Therefore, the HICA effects that attenuates NO and IL-6 overexpression might be contributed to improving skeletal muscle damage and maintaining the skeletal muscle mass for the athletes performing severe exercise, such as previously reported [2]. However, to clarify the contribution of the downregulation of basal protein synthesis by HICA to the expression of iNOS and/or IL-6, as well as atrogin-1 and MURF-1, further work, such as the quantification of mRNA, is needed.

One of the limitations of this study was that it was not possible to find the most upstream point of action of HICA. HICA is a hydroxy acid, and its presence has also been observed in muscle tissue [7]. Since the HICA content increased in the skeletal muscle after HICA administration in rats (unpublished data), it might enter into myotubes via a monocarboxylic acid transporter, such as the transporter specific for another Leu metabolite, β-hydroxy-β-methylbutyric acid [56,57]. On the other hand, it is possible that extracellular HICA could work as an agonist or antagonist of short-chain fatty acid receptors, such as the lactate receptor GPR81 that is expressed in muscle [58]. Therefore, further investigation to clarify the impact of these upstream points on the action of HICA in muscles is needed. 

In addition to this limitation, we admit that the concentrations of HICA used in this study are outside the range that can readily be adapted into the usual eating habits through the intake of fermented foods, because generally, fermentation does not result in the complete conversion of Leu to HICA [3]. Indeed, fermented skim milk that was used in our previous study [25] contained 580-μM HICA and 139-μM free Leu. This fermented skim milk administration (10-mL/kg body weight) in rats caused the HICA concentration in the portal vein to reach a maximum of 0.7 to 0.8 μM (unpublished data), but this concentration was drastically smaller than the HICA-exposed concentration in this study. The 15-mM concentration of HICA used here was also presumed to be higher than the concentrations achieved in the blood via the pure HICA administration performed in previous clinical and animal studies [2,14,15,16,59,60]. This level of HICA was similar to concentrations of lactate, which is released at high levels from muscles after rigorous exercise in humans [61] and, thus, might induce metabolic and/or environmental stress, such as high osmotic pressure. Therefore, further investigation into the effects of HICA on the skeletal muscle at more physiologically relevant doses is needed. 

We did not find evidence of the previously described systemic anti-inflammatory effects of HICA. We suggest, however, that HICA has the potential to improve systemic inflammation, because AMPK activation generally suppresses inflammation in several tissues [62]. The suppression of systemic inflammation leads to the maintenance of the muscle mass via increased energy efficiency, and it decreases the exposure of skeletal muscle to proinflammatory cytokines [63]. Thus, it will be important to investigate the effects of HICA on the other tissues (e.g., liver) and/or gut microbiota to clarify the systemic effects of HICA and to elucidate the differences between our findings and those from other animal or clinical trials.

**Figure A3:**
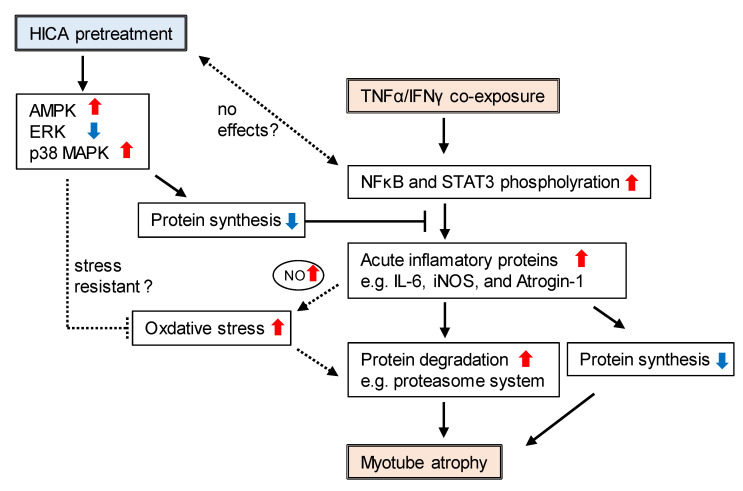
Schematic diagram of the HICA pretreatment effects on myotube atrophy under cachexic conditions. TNFα/IFNγ co-exposure increases NF-κB and STAT3 phosphorylation and, thus, accelerates the translocation of these molecules to the nucleus, resulting in the increasing transcription of their targets. Acute inflammatory proteins such as iNOS, IL-6, and atrogin-1 are increased directly or indirectly by NF-κB and STAT3 activation. These acute inflammatory proteins cause decreases of protein synthesis and increases of protein breakdown via the ubiquitin–proteasome system. As a result, the net balance of protein anabolism and catabolism becomes negative, leading to myotube atrophy. Moreover, iNOS overexpression should produce enough NO to enhance the oxidative stress. The HICA pretreatment cannot attenuate the NF-κB and STAT3 phosphorylation induced by cytokine exposure, although it activates the AMPK pathway. However, the HICA effect, which involves low basal protein synthesis via the inactivation of ERK signaling and activation of p38 MAPK and AMPK signaling should inhibit the translation of acute inflammatory proteins from their mRNA. Additionally, the HICA pretreated myotubes may experience resistance to stress via activation of the p38MAPK pathway, leading to a resistance to NO-induced oxidative stress. As a result, myotube atrophy induced by HICA pretreatment under the cachexia condition is caused via a mechanism that does not involve a direct impact on the NF-κB and/or STAT3 pathways.

In conclusion, under both normal conditions and a cachexic condition induced by the co-exposure to TNFα and IFNγ, direct HICA treatment decreased myotube protein synthesis, but it attenuated cachexic myotube atrophy by the suppression of protein breakdown. These effects might result from the relatively low basal protein synthesis caused by a pretreatment with HICA, and this led to the suppression of acute inflammatory responses such as iNOS and IL-6 overexpression, as well as the downregulation of the ubiquitin–proteasome system (summarized as a schematic diagram in Figure A3). Thus, our findings do not directly support the previous report that suggested that HICA increases protein synthesis in animal muscles [16]. However, the health benefits of HICA not only to skeletal muscle but, also, throughout the whole body should continue to be examined in order to better understand the value fermented foods have in contributing to the extension of a healthy life.
